# Parental homework involvement and students' mathematics achievement: a meta-analysis

**DOI:** 10.3389/fpsyg.2023.1218534

**Published:** 2023-07-13

**Authors:** Qiaodan Jiang, Li Shi, Donghui Zheng, Weijie Mao

**Affiliations:** College of Teacher Education, Ningbo University, Ningbo, China

**Keywords:** supportive parental homework involvement, intrusive parental homework involvement, students, mathematics achievement, meta-analysis

## Abstract

**Introduction:**

Given the importance of parent involvement to students' academic achievement, researchers have used a variety of methods to investigate the relationship between the two, but few focus on the relationship between parental homework involvement and students' achievement in a specific subject by using meta-analysis. This meta-analysis investigated the relationship between parent homework involvement and students' mathematics achievement from two dimensions: supportive (SPI) and intrusive parent homework involvement (IPI), along with their moderators.

**Methods:**

Accessed through Web of Science, Taylor and Francis Online, EBSCO, Springer Link, Elsevier, and ProQuest databases, a total of 20 empirical studies between 2005 to 2022, 41 independent effect sizes were included (*N* = 16,338). Effect size estimations were obtained by transforming Fisher's correlation coefficient. This study has conducted the heterogeneity tests of the magnitudes grouped according to different moderators, and investigated the publication bias that affects meta-analysis studies.

**Results and discussion:**

The results showed an overall positive link between SPI and students' mathematics achievement (*r* = 0.076, 95% CI = [0.037, 0.114]) and a negative link between IPI and students' mathematics achievement (*r* = −0.153, 95% CI = [−0.226, −0.079]). For the link of SPI and students' mathematics achievement, the effect sizes were (a) strongest when SPI was measured by autonomy support, followed by content support and provision of structure respectively; (b) stronger when students' mathematics achievement indicated by non-standardized measurement than standardized measurement. For the link of IPI and students' mathematics achievement, the effect sizes varied across grade level, strongest in high school, followed by middle school and lowest in primary school. These findings provide important implications for how to improve parental homework involvement practice to increase students' mathematics achievement.

## 1. Introduction

Homework as a valuable method of improving students' learning and academic achievement has been widely used across countries (Cooper et al., 2000; Trautwein, 2007; Trautwein and Lüdtke, 2009; Núñez et al., 2015; Fan et al., 2017; Šilinskas and Kikas, 2019b). Characterized by greater pressure and difficulty than other subjects, mathematics typically includes homework that requires help from parents (Kitsantas et al., 2011). Although a plethora of studies have proved that students' mathematics achievement was related to parental homework involvement (Patall et al., 2008; Dumont et al., 2012; Kikas et al., 2022), researchers have not reached a consistent conclusion on whether the relationship is positive or negative. Some argued that the two were positively related (e.g., Dumont et al., 2012; Gonida and Cortina, 2014; Lerner et al., 2021), while others found a negative link (e.g., Patall et al., 2008; Levpušček and Zupančič, 2009; Šilinskas et al., 2013; Šilinskas and Kikas, 2019b), making parental homework involvement became the most controversial one among all other types of parent involvement (Moroni et al., 2015).

Fiskerstrand (2022) recommended that it is essential to conduct a meta-analysis of the significance and causal–effect relationships at the indicator level between parental involvement and the mathematics outcome based on comparable quantitative methods. Thus, this study conducted a meta-analysis aimed at answering the following research questions:

(1) What is the relationship between parental homework involvement and students' mathematics achievement in basic education?
(2) Whether the relationship between parental homework involvement and students' mathematics achievement in basic education is influenced by a variety of moderating variables?

### 1.1. Parental homework involvement and students' mathematics achievement

Researchers have pointed out that the mixed conclusion was largely due to the types of parental involvement in homework (e.g., Ng et al., 2004; Pomerantz et al., 2007; Patall et al., 2008; Karbach et al., 2013; Gonida and Cortina, 2014; Suárez et al., 2014; Núñez et al., 2015), thus it is important to disentangle the different types of parental homework involvements, rather than to focus only on the quantity or frequency of involvement (Balli et al., 1997; Fan and Chen, 2001; Hoover-Dempsey et al., 2001; Pomerantz et al., 2007; Patall et al., 2008; Dumont et al., 2012).

Informed by the Self-Determination Theory (SDT) (Ryan and Deci, 2000, 2017), types of parental homework involvement were generally measured by two dimensions: supportive parental homework involvement (SPI) and intrusive parental homework involvement (IPI) (Moroni et al., 2015; Xu et al., 2018). According to SDT, parents' supportive involvement, such as autonomy support, has a positive influence on maintained intrinsic motivation, enhanced internalization, and greater psychological adjustment and wellbeing, whereas the parents' intrusive involvement, such as controlling, has a negative effect on children's important outcomes, leaving children feeling less engaged, being viewed by teacher as less competent, and becoming more physically aggressive over time. In addition, these general results held in young people from both individualistic and collectivist cultures. When the relationship was discussed from these two dimensions, the conclusion became clearer. Specifically, when parental homework involvement has been characterized as supportive (i.e., support of autonomy and provision of structure), a positive relationship between SPI and students' achievement has been found (Cooper et al., 2000; Pomerantz et al., 2005). However, IPI (i.e., controlling or monitoring) was generally associated with negative or null outcomes of student learning and achievement (Ng et al., 2004; Brown, 2005; Pomerantz et al., 2007; Patall et al., 2008; Dumont et al., 2014; Gonida and Cortina, 2014; Moè et al., 2018; Xu et al., 2018; Šilinskas and Kikas, 2019b).

In this meta-analysis, we expect to get a conclusion consistent with the abovementioned research and propose the following hypotheses:

**H1:** Students' mathematics achievement is positively related to supportive parental homework involvement (SPI).
**H2:** Students' mathematics achievement is negatively related to intrusive parental homework involvement (IPI).

### 1.2. Potential moderators

Findings from previous studies on the relationship between parental homework involvement and students' academic achievement are inconclusive. On the one hand, the insufficient sample size for each separate study may be the reason for the mixed results. On the other hand, results vary depending on factors such as the different dimensions of the parental homework involvement measured (e.g., parent homework control vs. parents homework support; Kikas et al., 2022); different participants' types (e.g., students vs. parents vs. teachers; Erdem and Kaya, 2020); different measuring tools of students' mathematics achievement (e.g., non-standardized measurement vs. standardized test; Jeynes, 2005; Castro et al., 2015); different demographics characteristics such student grade level (e.g., primary school vs. Middle school vs. High school; Núñez et al., 2015), region and culture (e.g., minority vs. white students; Jeynes, 2005) among studies. Meanwhile, different study attributes, such as the type and year of publications, may also lead to inconsistent research results. Therefore, this meta-analysis addressed the small sample size issue and tested the moderating effects from three aspects: measurement tools, demographic variables, and study attributes, in order to model different results across studies.

#### 1.2.1. Measuring tools

##### 1.2.1.1. Type of SPI and IPI

How SPI and IPI were measured may lead to distinctive results. By comparing the questionnaires of SPI and IPI in past research, we found that SPI may measure several typical sub-types, including autonomy support, content support, and provision of structure, while IPI was generally measured by parental control and interference. Specifically, questions such as “My parents convey confidence in my ability to do math homework assignments (Xu and Corno, 2022); When my parents help me with my school work, they always encourage me to find the correct answer by myself (Karbach et al., 2013)” were used to measure parent autonomy support, which can be defined as “allowing children to explore their environment, initiate their own behavior, and take an active role in solving problems” (Pomerantz et al., 2007). SDT indicated that when acting with autonomy, behaviors are engaged wholeheartedly, whereas one experiences incongruence, and conflict when doing what is contrary to one's volition. What is more important in most settings having support for autonomy as a contextual factor plays a critical role in allowing individuals to actively satisfy all of their needs—to gravitate toward, make relevant choices in relation to, and employ optimizing strategies for satisfying each basic need (Ryan and Deci, 2017). In other words, autonomy support is seen as the most critical aspect of the satisfaction of human psychological needs. Thus, it is believed that when parental homework involvement is measured by autonomy support, the largest correlation should be discovered in the SPI-students' mathematics achievement link.

Questions such as “My parents help me with math if I ask them; I can always ask my parents if I don't understand something in math” were used to measure content support, another sub-type of SPI, referring to the extent to which parents provide direct help on homework when asked by children (Xu et al., 2018; Xu and Corno, 2022). By being available for help if needed, content support tends to increase students' sense of autonomy, sense of competence, and persistence in learning (Moorman and Pomerantz, 2008). Nevertheless, Xu et al. (2018) revealed that as compared with parental autonomy support, parental content support may backfire even when asked by children. Since parental content support may lead to a sense of incompetence in children, and when asked by children for content support, many parents may find it difficult to withdraw their support as children become more competent and are well on their own. Therefore, we speculate that when parental homework involvement is measured by content support, it may also have a positive impact on students' math achievement, although this correlation may not be as significant as the parent autonomy support students' math achievement link.

Questions such as “Do you provide incentives for your child to finish his/her mathematics homework (O'Sullivan et al., 2014); whether the television was on or off when their child did homework (Cooper et al., 2000)” were used to measure “provision of structure”, referring to the degree of parents provide clear and consistent guidelines and follow through on contingencies for their children's homework (Cooper et al., 2000). SDT indicated that the provision of structure supports one's competence needs. The need for competence is evident as an inherent striving, manifested in curiosity, manipulation, and a wide range of epistemic motives (Deci and Moller, 2005). In this way, parental provision of structure may enhance children's sense of competence, believing that they can exert a positive influence on their grades and other academic outcomes (O'Sullivan et al., 2014). Nevertheless, Wang and Cai (2017) indicated that the impact of the parental provision of structure on students' math achievement may largely depend on how students perceive their parents' behavior. For example, parental provision of structure is positively associated with students' academic performance in China, given that Chinese children may perceive parental provision of structure as an act of love. Thus, we speculate that when parental homework involvement is measured by the provision of structure, it may have a positive impact on students' math achievement, provided that students view it as a supportive involvement.

**H3-a:** The positive correlation is strongest when SPI was measured by autonomy support, followed by content support and provision of structure, respectively.

For IPI, questions such as “Me doing homework is very important to my parents; My parents scold and punish me if I don't do all the homework (Núñez et al., 2015); I insisted my child do things in my way when it came to doing his/her math homework (Wu et al., 2022)” were used to measure parent homework controlling, which can be defined as “control and pressure on student to complete assignments” (Šilinskas and Kikas, 2019b). Questions such as “My parents often interfere when I'm doing my math homework; When I'm doing math homework, my parents ask if I need help (Kikas et al., 2022)” were used to measure parental interference which refers to parents' tendency to solve the students' homework although the student has not asked for it or interrupting student in their homework (Moroni et al., 2015). It has been shown that parental control decreases students' sense of autonomy, sense of competence, and effort in challenging learning situations (Pomerantz et al., 2007). On the other hand, interference was the most damaging type of parental homework involvement because it undermined mastery goal orientation and reduced perceived competence (Gonida and Cortina, 2014). Thus, we generate the following hypothesis:

**H3-b:** The negative correlation is strongest when IPI was measured by interference, followed by controlling.

##### 1.2.1.2. Questionnaire reporter

Parental homework involvement questionnaire reporters might have an impact on the parental homework involvement-students' math achievement link, as parents' and students' perceptions regarding parental homework involvement may differ. It is likely that students' perceptions of parental homework involvement are more real or “knowable” to them than the actual nature or extent of parents' behavior related to homework (Grolnick and Slowiaczek, 1994; Hoover-Dempsey et al., 2005). Studies have also pointed out that students' interpretations of parental involvement often shape their responses to that involvement and are therefore more closely related to their development than parents' actual behavior (Schaefer, 1965; Grolnick et al., 1991; Hoover-Dempsey et al., 2005). Based on that, we can speculate as follows:

**H4:** When the parental homework involvement questionnaire is reported by students, the relationship between parental homework involvement and students' math achievement is stronger than when reported by parents themselves.

##### 1.2.1.3. Mathematics achievement indicator

Different indicators of students' mathematics achievement may also yield different results. Andrews and Harlen (2006) suggested that various assessments of academic achievement could present problems during the synthesis stage of the study that would challenge the usefulness of the findings. A meta-analysis further revealed that “the manner of assessing student scholastic performance did not seem to impact the existence of the relationship between parental involvement and academic achievement. It did, however, affect the strength of that relationship” (Wilder, 2014). Compared to standardized tests that typically have tighter confidence intervals and smaller standard deviations for the test scores, non-standardized measurement can be easily influenced by many factors or biases of the assessor. Since Jeynes (2005) revealed that the teacher as a significant person in rating students' mathematics performance is likely to be influenced by a high degree of parent involvement. It is possible that when students' mathematics achievement is reported by non-standardized measurement, larger parental homework involvement-students' mathematics achievement links may find. Given this, we propose the following hypothesis:

**H5:** In both SPI-students' math achievement and IPI-students' math achievement link, students' mathematics achievement reported by non-standardized measurement have larger links than those reported by standardized tests.

#### 1.2.2. Demographic variables

##### 1.2.2.1. Culture

Differences in culture might also drive inconsistent results. Since the existing research on the relationship between parental homework involvement and students' mathematics achievement was mainly conducted in a certain area, it remained a research gap to investigate the potential moderating effect of cultural background, so we test it in this meta-analysis. Danişman (2017), pointed out that the moderating effect of culture was statistically significant in the parent involvement and students' achievement link (*Q* = 5.382, *p* < 0.05). Specifically, parents from collectivist countries (*r* = 0.43) had a stronger effect on student achievement than those from individualist (*r* = 0.30) countries. According to Hofstede (1991) cultural dimensions theory, people in collectivist cultures feel as if they belong to larger in-groups or collectives which care for them in exchange for loyalty. As a result, a collectivist culture is especially likely to emphasize the importance of social harmony, respectfulness, and group needs over individual needs. Thus, the relationship between parents and children might be closer in collectivist cultures, and parental homework involvement may have a greater impact on students' math achievement. On the contrary, people who live in individualist cultures tend to believe that independence, competition, and personal achievement are more important. Children tend to complete their homework independently. Thus, parental homework involvement may not have a significant impact on students' math achievement.

**H6:** Compared with individualism, the correlation between parents' homework involvement and students' math achievement under the collectivism culture is stronger.

##### 1.2.2.2. Grade level

Past studies suggested that students' grade levels moderated the link between parental homework involvement and students' achievement (e.g., Skaliotis, 2010). Since younger students appear to have less developed study habits, parental homework involvement has been found to have desirable effects on elementary school students (Dufresne and Kobasigawa, 1989). However, others found contradictory results that the relationship between perceived parental homework involvement and academic achievement was stronger in middle high school and high school than in elementary school (Núñez et al., 2015). The inconsistent conclusion largely fails to consider the type of parental homework involvement. We speculate that lower-grade students often lack the ability to self-control and self-management, and have not formed good learning habits or strategies yet. At this stage, parental supportive homework involvement will have the strongest effect on improving their academic achievement. Furthermore, younger students, who have not yet developed independent personalities, rely more on their parents' help, therefore might have a greater tolerance for parental control or interference in homework. However, students in middle and high school have gradually developed an independent learning style, and they no longer require much supportive homework involvement from their parents, making the correlation between SPI and math achievement weakened. Furthermore, puberty sharply distinguishes middle and high school students from other students, by changing their brains yielding greater emotional intensity (Nelson et al., 2012). SDT also revealed that psychological needs, satisfactions, and frustrations vary within persons over time. Therefore, IPI may cause their extremely strong resistance, and eventually lead to a stronger negative impact on middle and high school students' math achievement. We generate the following hypothesis, hoping to adjudicate these mixed results:

**H7-a:** As students' grades increase, the correlation between SPI and students' math achievement gradually weakens.
**H7-b:** As students' grades increase, the correlation between IPI and students' math achievement gradually strengthens.

#### 1.2.3. Study attributes

##### 1.2.3.1. Publication type

Publication type may affect the relationship between parental homework involvement and students' mathematics achievement. It has been well established that journals are more likely to publish significant findings than non-significant findings (Card, 2015), and the non-significant results are usually excluded from quantitative reviews of research results. Therefore, the effect size may be larger in journal articles than in dissertations.

##### 1.2.3.2. Publication year

The publication year of studies may moderate the relationship between parental homework involvement and students' mathematics achievement. From the perspective of technological progress, the rapid development of information technology has brought a new look to student mathematics learning. Using online homework tools in mathematics learning has thus become a new phenomenon that complements traditional homework (Sarmiento, 2017). Though such web-based mathematics homework can help students obtain skills that lessen anxiety and raise students' consciousness in the learning process (Albelbisi, 2019), it often requires more parental involvement as well. Meanwhile, global, national, and local policies also started to promote the importance of parent education involvement and advocate for a greater role of parents in education in order to enhance the academic achievement of their children (Englund et al., 2004). Therefore, parental homework involvement behavior may increase over time, and the relationship between parental homework involvement and students' mathematics achievement might become stronger.

### 1.3. This study

In this meta-analysis, we aim to synthesize the results of previous studies testing the impact of SPI and IPI on students' mathematics achievement and to identify the potential factors that moderate it. First, we sum up the overall effect size of the relationship between SPI and students' mathematics achievement, IPI, and students' mathematics achievement, respectively. Next, we explore whether this relationship differs across measuring tools (type of SPI/IPI, questionnaire reporter, mathematics achievement indicator), demographics (culture and grade level), and study attributes (publication type and year) by testing moderators.

## 2. Research methods

### 2.1. Literature search and screening

This study mainly uses electronic retrieval to collect journals and doctoral dissertations about the relationship between parental homework involvement and students' mathematics achievement (Unpublished documents such as government documents and conference papers are not included in the search scope) between June 2005 (No earlier studies of parental homework involvement and student's mathematics achievement) to December 2022. We searched the following databases: Web of Science, Taylor and Francis Online, EBSCO, Springer Link, Elsevier, and ProQuest databases. Meanwhile, Google Scholar was used to assist with retrieval.

The literature search has gone through two rounds of procedures. The first round was extensive searching through keywords compilation. During the search process, it was found that there were few relevant articles about the relationship between parental homework involvement and students' mathematics achievement. Most of the studies on the relationship between them were included in a broader scope of “parent involvement and students' academic achievements” for discussion. In order to collect articles as much as possible, we took the following as the retrieval formula, combining three retrieval fields of subject, title, and full text:

(parent involvement OR parent engagement OR parent participation OR parent help) AND (academic achievements OR academic attainment OR academic outcomes OR academic scores OR academic grades).

A total of 338 articles were obtained in the first round of large-scale retrieval. The second round of retrieval was based on citation backtracking. By tracking the references and cited articles of the articles obtained from the first round, 96 articles were obtained in this round. After deleting 25 repetitive articles, 409 articles were obtained in two rounds.

**Figure 1:**
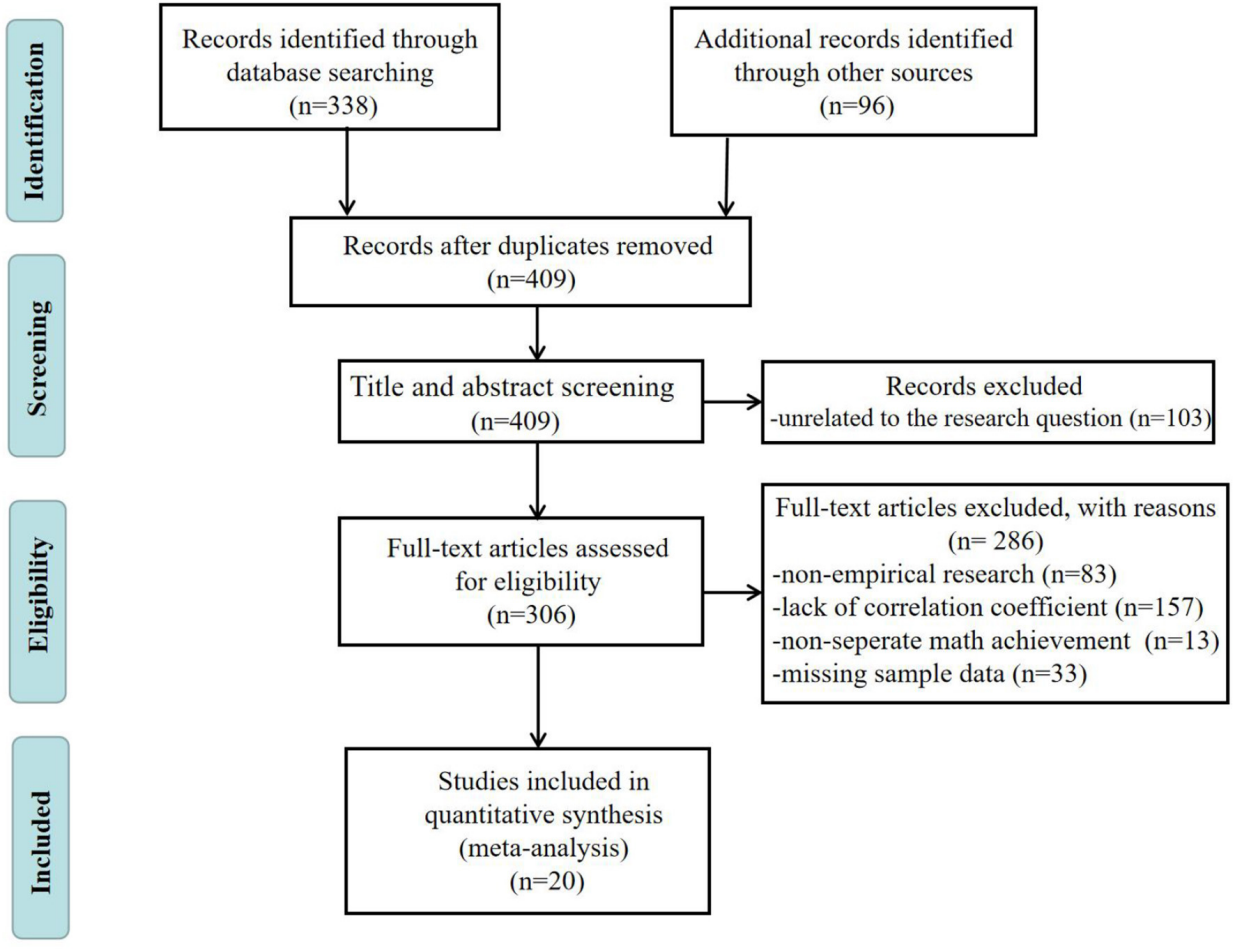
Flow diagram of literature search and study inclusion criteria.

Subsequently, we began two rounds of screening for these 409 articles. By reading the titles and abstracts, 103 articles unrelated to the research question were excluded in the first round of screening. The second round of screening was conducted by reading the full text of the remaining 306 articles. The inclusion criteria for this round of screening are as follows (see Figure 1 for a flow chart of the article selection process): (1) only empirical studies are included; (2) the Pearson's product-moment correlation coefficient *r* between parental homework involvement and students' mathematics achievement is clearly reported; (3) it reports the measuring tool of students' mathematics achievement (The mathematics achievement here do not include comprehensive achievement including math, such as GPA, composite scores of language and math, etc.); and (4) it reports the sample size. By reading the abstract and full text while screening according to the above criteria, 20 articles published between 2005 and 2022 met the requirements and were finally included in the study.

### 2.2. Coding variables

The selected articles were coded according to the constituent elements, and each independent sample was coded only once (See Table 1 for coding results).

1. References: Author, Year of publication (if the same study contains multiple results, it shall be distinguished by serial number).
2. Type of SPI/IPI^1^: Supportive (Autonomy Support, Content Support, Provision of Structure); Intrusive (Controlling, Interference).
3. Questionnaire reporter: Students; Parents.
4. Mathematics achievement indicator^2^: Standardized measurement; Non-standardized measurement.
5. Culture: Individualist; Collectivist (Refer to the evaluation results of Hofstede Cultural Guide for judgment of cultural background of different countries/regions: https://www.hofstede-insights.com/).
6. Grade level: Primary school; Middle school; High school; Mixed.
7. Publication type: Journal; Doctoral dissertation.

**Table 1:** Characteristics of the 41 studies in the meta-analysis.

**References**	**Type of SPI/IPI^a^**	**Questionnaire reporter^b^**	**Math achievement indicator^c^**	**Culture^d^**	**Grade level^e^**	**Publication type^f^**	**r**
**Supportive parental homework involvement (SPI)**							
Dumont et al. (2012)^1^	2	1	2	1	2	1	0.050
Karbach et al. (2013)	1	1	2	1	2	1	0.070
Kikas et al. (2022)^1^	2	1	1	1	1	1	−0.050
Kikas et al. (2022)^3^	2	1	1	1	2	1	0.050
Lerner et al. (2021)	1	1	2	1	1	1	0.140
Núñez et al. (2015)^2^	2	1	2	1	1	1	0.140
Núñez et al. (2015)^4^	2	1	2	1	2	1	0.150
Núñez et al. (2015)^6^	2	1	2	1	3	1	0.150
Núñez et al. (2017)^2^	2	1	2	1	4	1	0.150
O'Sullivan et al. (2014)^1^	3	2	2	1	2	1	0.240
O'Sullivan et al. (2014)^2^	1	2	2	1	2	1	0.190
Nwokedi (2020)^1^	3	1	1	1	2	2	−0.223
Nwokedi (2020)^2^	3	1	1	1	1	2	0.025
Nwokedi (2020)^3^	3	1	1	1	2	2	−0.179
Šilinskas and Kikas (2019a)^2^	2	1	1	1	1	1	−0.090
Šilinskas and Kikas (2019a)^4^	2	1	1	1	2	1	0.050
Viljaranta et al. (2018)^2^	1	2	1	1	1	1	0.210
Wu et al. (2022)^2^	1	2	1	1	1	1	0.060
Xu and Corno (2022)^1^	2	1	1	2	2	1	0.020
Xu and Corno (2022)^2^	1	1	1	2	2	1	0.110
Xu et al. (2018)^1^	1	1	1	2	2	1	0.230
Xu et al. (2018)^2^	2	1	1	2	2	1	−0.110
**Intrusive parental homework involvement (IPI)**							
Brown (2005)	4	2	1	1	4	2	−0.053
Dumont et al. (2012)^2^	5	1	2	1	2	1	−0.160
Kikas et al. (2022)^2^	5	1	1	1	1	1	−0.270
Kikas et al. (2022)^4^	5	1	1	1	2	1	−0.200
Núñez et al. (2015)^1^	4	1	2	1	1	1	−0.260
Núñez et al. (2015)^3^	4	1	2	1	2	1	−0.320
Núñez et al. (2015)^5^	4	1	2	1	3	1	−0.360
Núñez et al. (2017)^1^	5	1	2	1	4	1	−0.050
Purnomo et al. (2022)	5	1	1	2	1	1	0.757
Retanal et al. (2021)^1^	4	2	2	1	4	1	−0.210
Retanal et al. (2021)^2^	5	2	2	1	4	1	−0.170
Rogers et al. (2009)	4	1	2	1	1	1	−0.020
Šilinskas and Kikas (2019a)^1^	5	1	1	1	1	1	−0.390
Šilinskas and Kikas (2019a)^3^	5	1	1	1	2	1	−0.240
Šilinskas and Kikas (2019b)	5	1	1	1	1	1	−0.390
Šilinskas et al. (2013)	4	2	1	1	1	1	−0.220
Viljaranta et al. (2018)^1^	4	2	1	1	1	1	−0.220
Wachiya Indimuli (2022)	4	2	1	1	1	1	0.360
Wu et al. (2022)^1^	4	2	1	1	1	1	−0.160

^a, 1^Autonomy Support; ^2^Content Support; ^3^Provision of Structure; ^4^Control; ^5^Interference. ^b, 1^Students; ^2^Parents. ^c, 1^Standardized measurement; ^2^Non-standardized measurement. ^d, 1^Individualist; ^2^Collectivist. ^e, 1^Primary School; ^2^Middle School; ^3^High school; ^4^Mixed. ^f, 1^Journal; ^2^Doctoral dissertation.

In order to ensure the coding reliability, two researchers who studied and regularly run meta-analyses coded the included articles separately. Cohen's kappa coefficient was used to analyze the consistency of the two researchers coding results for the two moderators (types of SPI/IPI, mathematics achievement indicators) that may have different opinions. Results showed that Cohen's kappa coefficient was 0.969 (*p* < 0.0001) and 0.945 (*p* < 0.0001), respectively, indicating that there was a strong consistency between them. Then, the two researchers discussed their disagreements and agreed on the final codes *via* consensus.

### 2.3. Assessment of study quality

The methodology quality of included studies was assessed by two independent reviewers using the standardized critical appraisal instruments prepared by the Joanna Briggs Institute (JBI). For cross-sectional surveys, the JBI Critical Appraisal Checklist for prevalence studies was used. This tool comprised nine questions, and studies that obtained five or more “Yes” ratings out of nine were included in the review (Munn et al., 2015). For longitudinal studies (e.g., Šilinskas et al., 2013; Viljaranta et al., 2018; Šilinskas and Kikas, 2019a,b; Kikas et al., 2022), JBI Critical Appraisal Checklist for cohort studies was used. This tool comprised eleven questions, and studies that obtained <6 “Yes” scores were excluded. The final score consistency of the two independent reviewers was 0.85. All 20 studies met the inclusion standard, indicating that the quality of the studies included in this study met the analysis requirements.

### 2.4. Effect size calculation

In this meta-analysis, data were analyzed using Comprehensive Meta Analysis 3.0, and Pearson's product–moment correlation coefficient *r* was used to calculate the effect size. First, we extracted the initial effect size in each study, that is, the correlation coefficient *r* between parents' homework involvement and students' mathematics achievement. Then, Fisher's z-transformation was applied to *r*, weighted based on the sample size with 95% confidence intervals: Z = 0.5^*^ ln [(1 + r)/(1 – r)], where the variance of Z is V_Z_ = 1/n−3 and the standard deviation of Z is SE_Z_ = square root of (1/n−3).

### 2.5. Data processing and analysis

Homogeneity tests determined whether each result was significantly different from the overall effect size, which informs the selection of a fixed-effect model vs. a random-effect model. If a homogeneity test shows that the effect size is homogeneous, a fixed-effect model is used. If it indicates significantly large heterogeneity in the effect size, a random-effect model is used. In addition, large heterogeneity suggests potential moderation effects (Lipsey and Wilson, 2001; Card et al., 2010).

### 2.6. Sensitivity analysis

We conducted a cumulative analysis to assess if the effect size estimate stabilizes with the inclusion of studies. If any new study produces a sudden shift as the volume of data accumulates, then there might exist a bias (Borenstein et al., 2009).

### 2.7. Evaluation of publication bias

We assessed the risk of publication bias through funnel plot and Egger's linear regression method to determine whether potential bias affects the validity and robustness of research results under different circumstances. CMA software is used to draw funnel plots that can visually identify deviations, and Egger's regression method is used to quantify the asymmetry of funnel plots. The assumption is that, without publication bias, the scattered points representing each study will be symmetrically distributed on both sides of the average effect quantity, and the intercept of Egger's regression is close to 0 and not significant (Egger et al., 1997). On the contrary, when the scatter points are asymmetric and the *p*-value of Egger's test is <0.05, it indicates the existence of publication bias.

## 3. Results

### 3.1. Effect size and homogeneity tests

**Figure 2:**
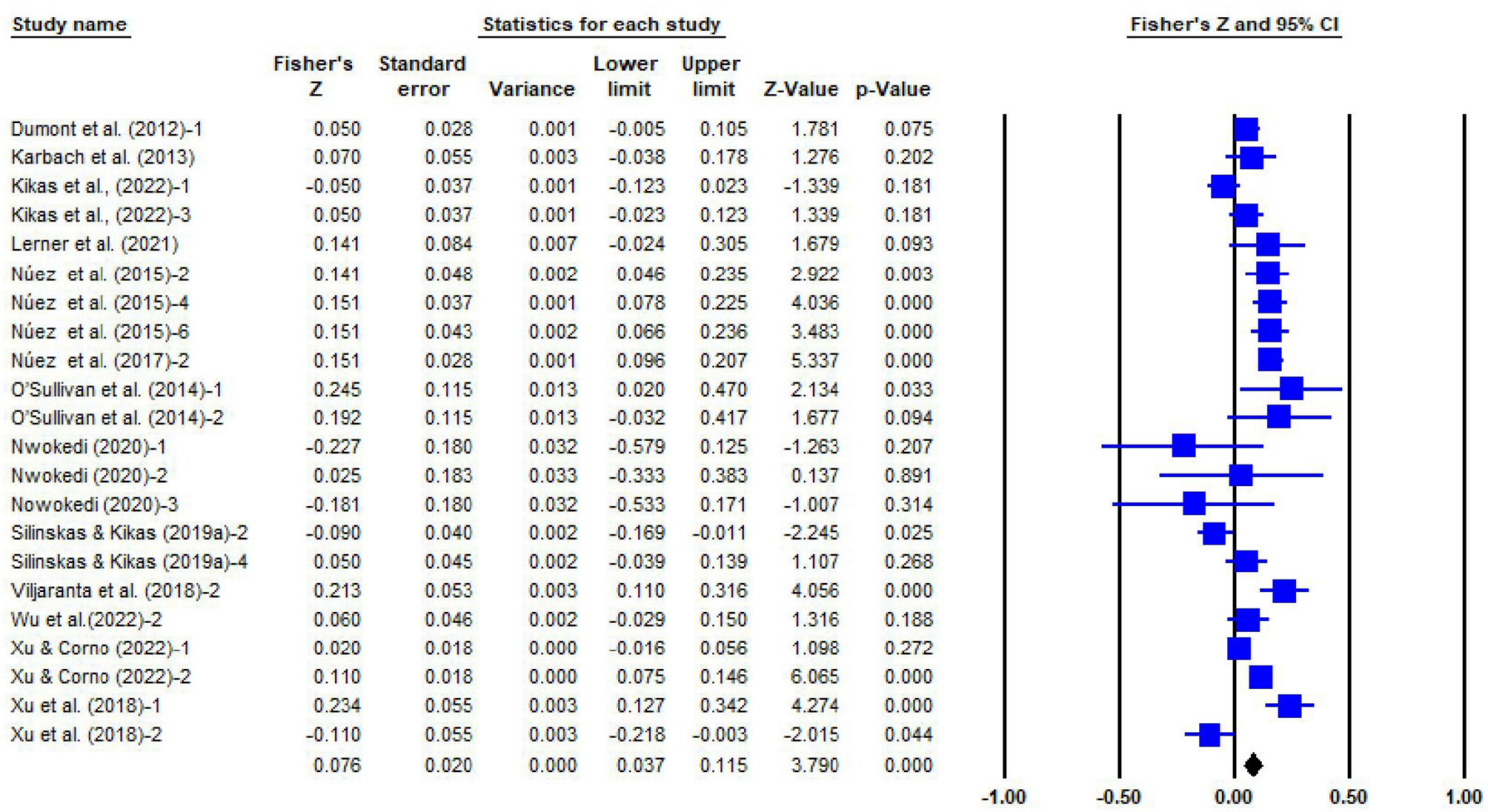
Forest plot for the random-effects model of 22 studies (SPI).

**Figure 3:**
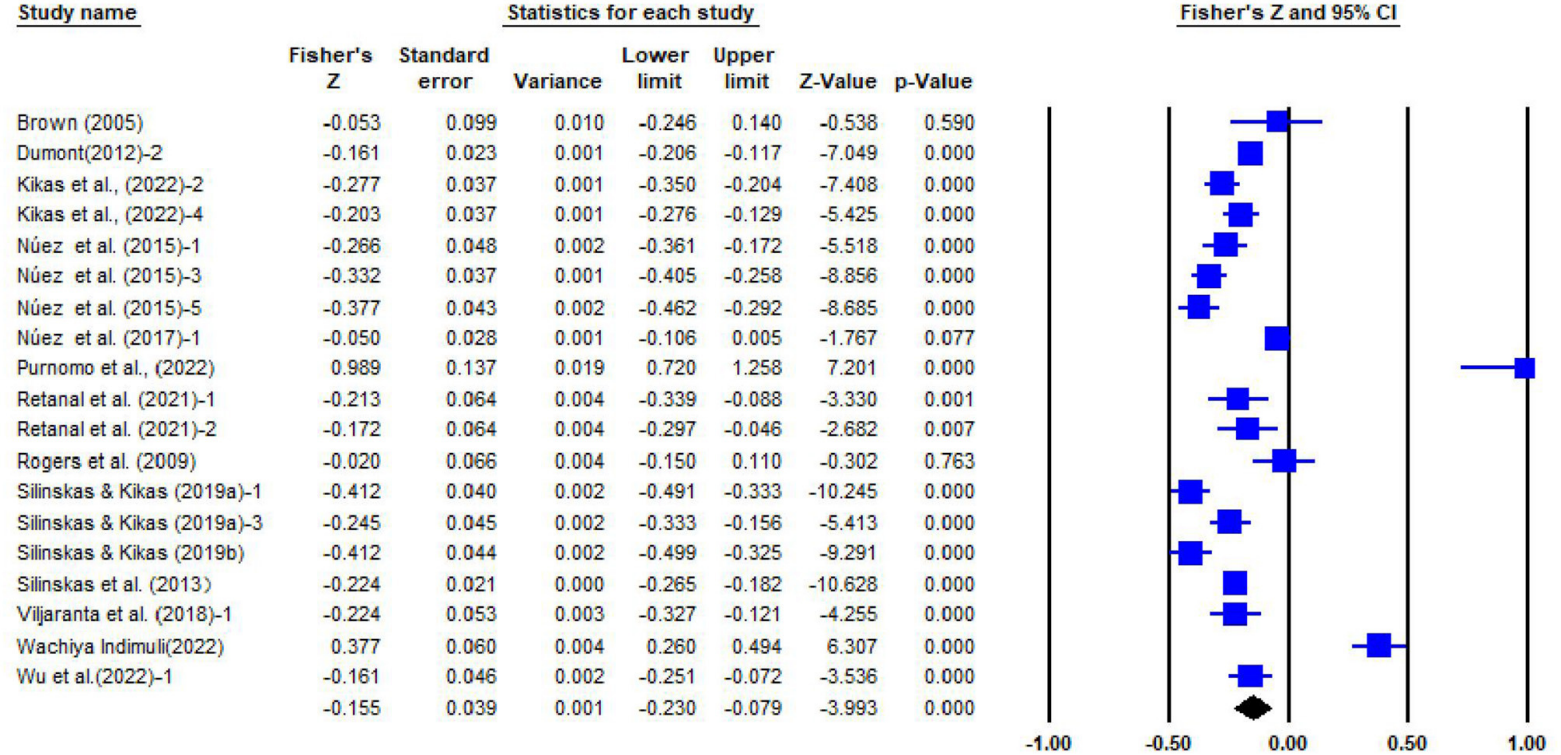
Forest plot for the random-effects model of 19 studies (IPI).

This meta-analysis of 20 articles and 41 independent effect sizes had 16,338 participants. The sample sizes of the studies ranged from 33 to 3,018. The average sample size is about 583, and the time span is 2005–2022. As illustrated in the Table 2 and forest plot of SPI and IPI (see Figures 2, 3), the homogeneity tests for 22 independent samples of SPI and 19 independent samples of IPI both showed substantial heterogeneity among the selected studies (*Q*_SPI_ = 94.391, *df* = 21, *p* < 0.0001; *Q*_IPI_ = 297.629, *df* = 18, *p* < 0.0001) and likely moderation effects. Meanwhile, $I_{\text{SPI}}^{2}$ = 77.752%, $I_{\text{IPI}}^{2}$ = 93.952%, both are larger than 75%, indicating that there were variables moderating the relationship between parental homework involvement and students' math achievement (*I*^2^values: 25% [low], 50% [medium], 75% [high]; Higgins and Thompson, 2002), so a random-effect model was used.

**Table 2:** Random-effect model of the correlation between parental homework involvement and students' mathematics achievement.

	***k***	**Mean *r***	**95% CI for *r***	**Homogeneity test**			**Tau-squared**		**Test of null (two-tailed)**
				***Q(g)***	***p***	***I** ^2^*	***Tau** ^2^*	***Tau***	***Z-Value***
SPI	22	0.076	[0.037, 0.114]	94.391	0.000	77.752	0.006	0.074	3.790^***^
IPI	19	−0.153	[−0.226, −0.079]	297.629	0.000	93.952	0.025	0.159	−3.993^***^

^***^p < 0.001. SPI, Supportive parental homework involvement, IPI, Intrusive parental homework involvement; *k* is the sample size of the independent study.

The random-effect model showed a significant positive correlation between SPI and students' math achievement (*r* = 0.076, 95% CI = [0.037, 0.114]), and a significant negative correlation between IPI and students' math achievement (*r* = −0.153, 95% CI = [−0.226, −0.079]).

### 3.2. Sensitivity analysis

**Figure 4:**
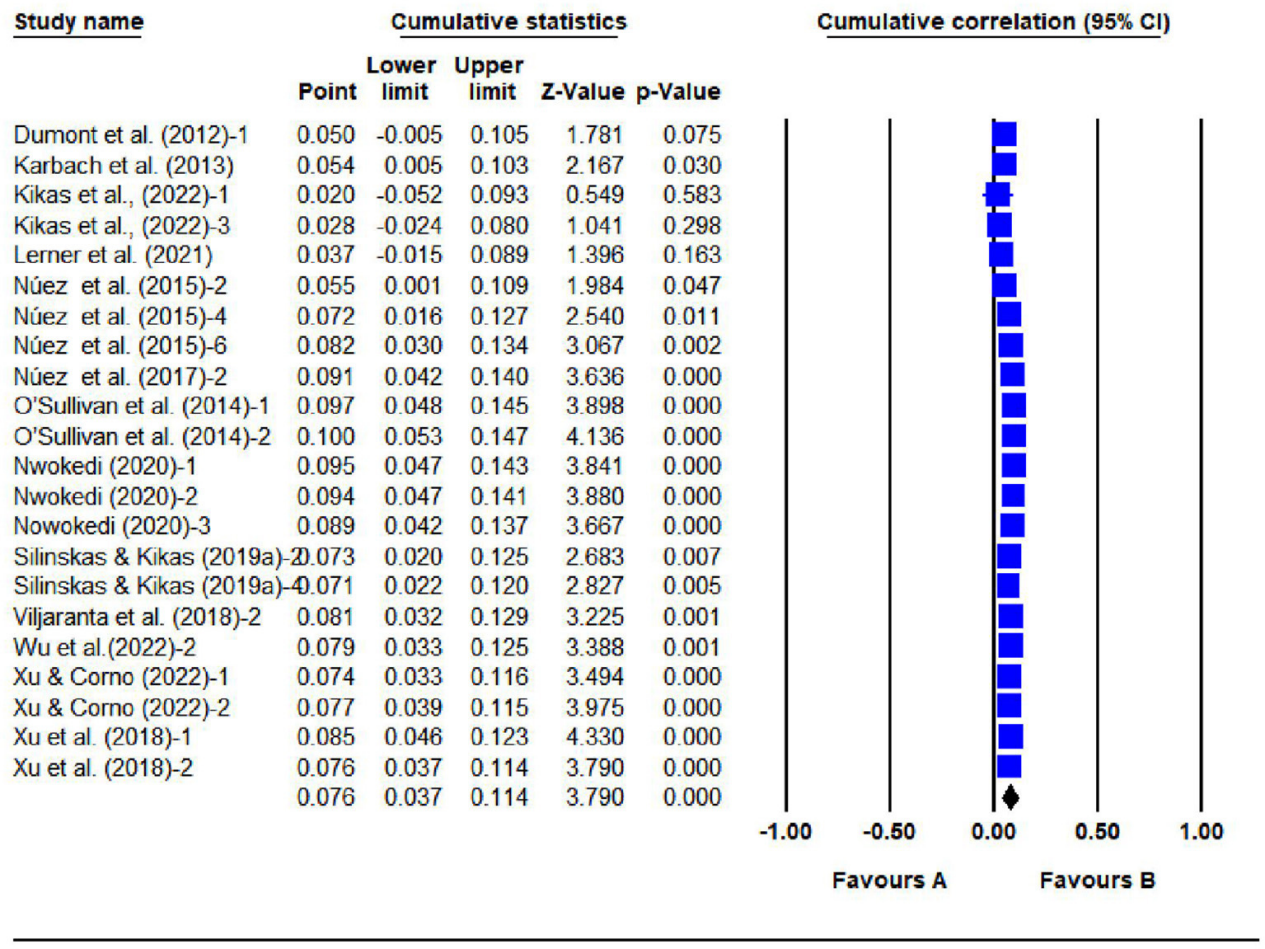
Cumulative analysis results for 22 SPI studies.

**Figure 5:**
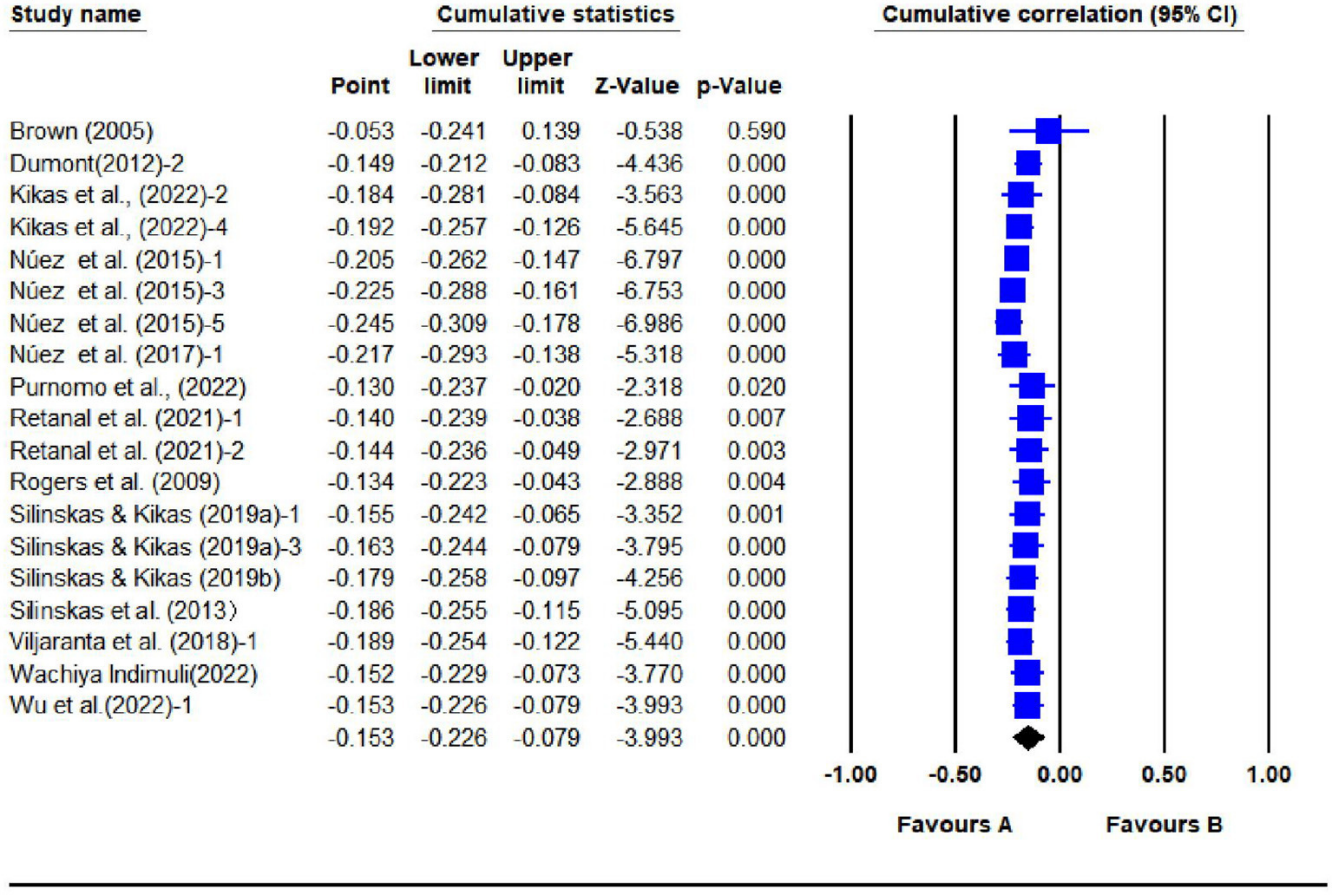
Cumulative analysis results for 19 IPI studies.

As is shown in Figures 4, 5, the effect size tended to stabilize and the confidence intervals tended to narrow as studies were added to the analysis, which suggests that the results were robust to our assumptions.

### 3.3. Publication bias tests

**Figure 6:**
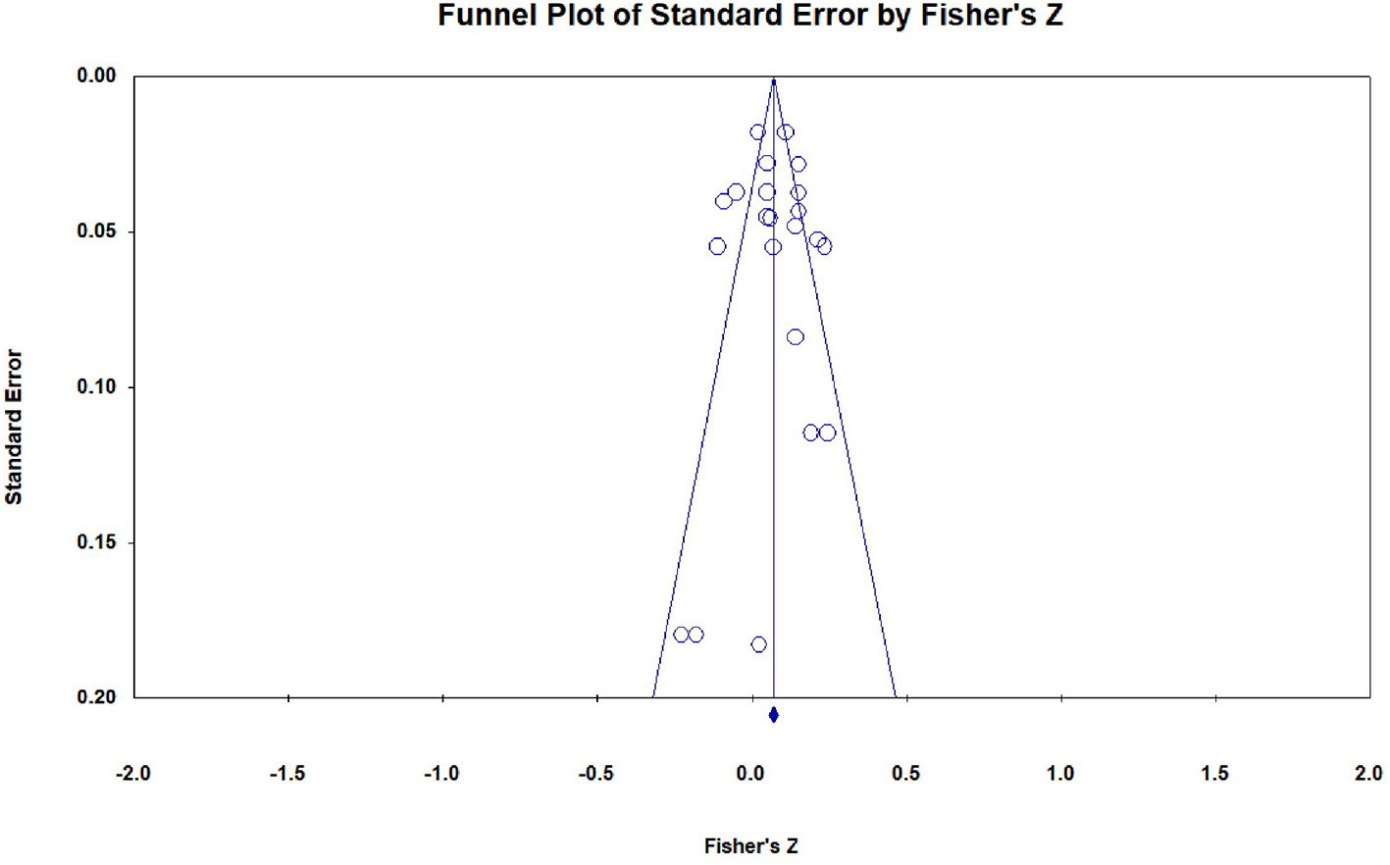
Funnel plot of effect sizes of the correlation between SPI and students' mathematics achievement.

**Figure 7:**
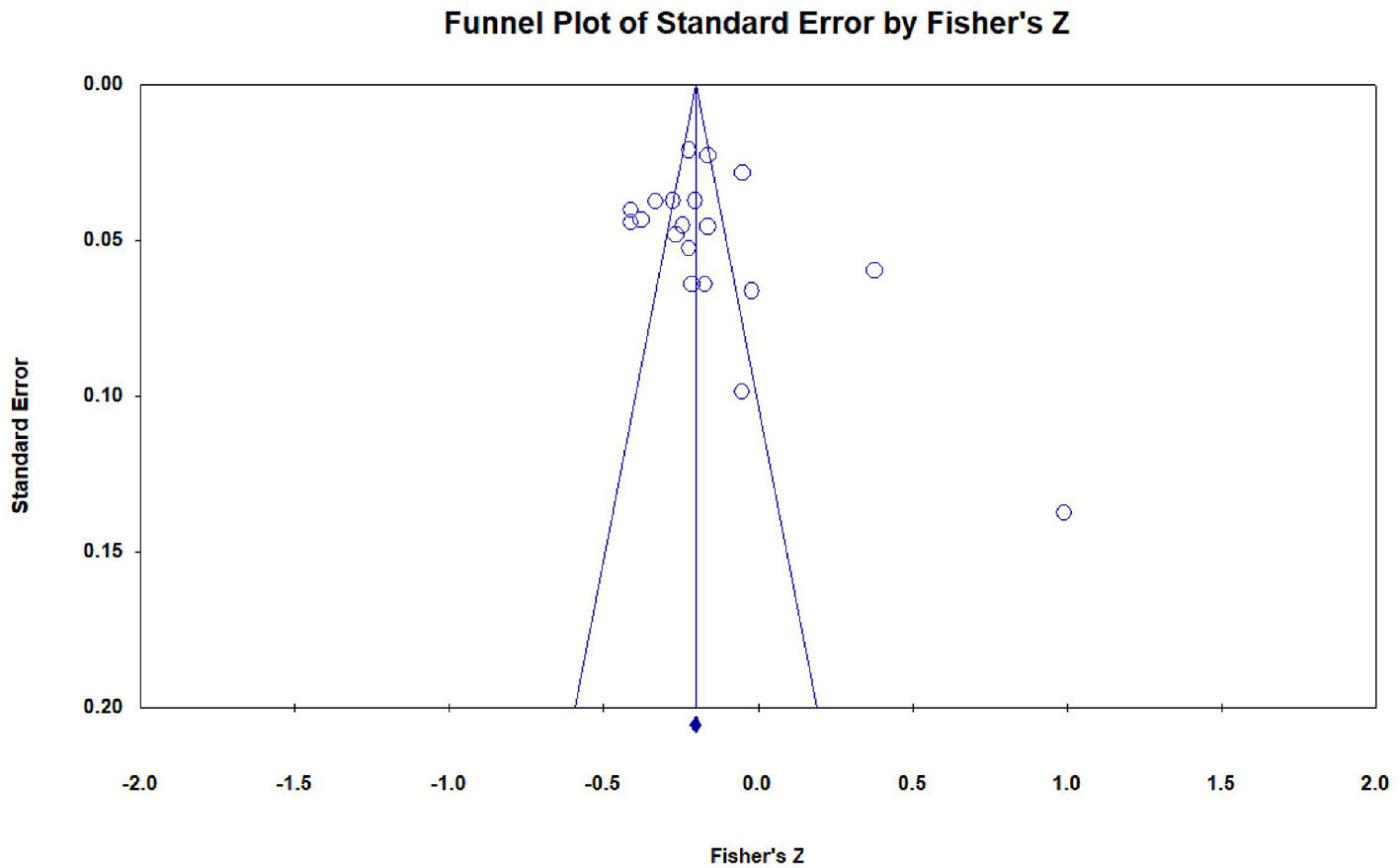
Funnel plot of effect sizes of the correlation between IPI and students' mathematics achievement.

As shown in Figures 6, 7, there was no obvious asymmetry in the funnel plots, which indicated that there was no publication bias. In addition, Egger's regression test showed that t_SPI(22)_ = 0.092, *p* = 0.928; t_IPI(19)_ = 1.169, *p* = 0.258, which further verified that there was no potential publication bias in the data set. Therefore, the abovementioned tests support that the effects included in this study have no publication bias.

### 3.4. Moderator analysis

We used a meta-analysis of variance to test the potential moderate effect of six categories of variables: type of SPI/IPI, questionnaire reporter, mathematics achievement indicator, culture, grade level, and publication type. Meanwhile, meta-regression analysis was used to test the potential moderating effect of the publication year (see Tables 3, 4).

**Table 3:** Correlation between parental homework involvement and students' mathematics achievement: Univariate analysis of variance for the moderator variables (categorical variables).

	**Between-group effect (*Q_*BET*_*)**	***k***	**Mean *r***	**SE**	**95% CI for *r***	**Homogeneity test within each group (*Q_*W*_*)**	***I^2^***
**Supportive parental homework involvement (SPI)**							
**Measuring tools**							
**Type of SPI**	6.216^*^						
Autonomy support		7	0.133	0.002	[0.084, 0.181]	10.759	44.234
Content support		11	0.049	0.004	[−0.002, 0.099]	59.650^***^	83.236
Provision of structure		4	−0.009	0.049	[−0.243, 0.227]	6.914	56.609
**Questionnaire reporter**	0.084						
Students		18	0.062	0.003	[0.020, 0.104]	83.367^***^	79.608
Parents		4	0.156	0.008	[0.058, 0.252]	6.047	50.389
**Math achievement indicators**	6.830^**^						
Standardized measurement		13	0.036	0.005	[−0.019, 0.091]	66.662^***^	81.999
Non-standardized measurement		9	0.123	0.001	[0.087, 0.159]	11.271	29.024
**Demographics**							
**Culture**	0.088						
Individualism		18	0.079	0.003	[0.033, 0.125]	61.717^***^	72.455
Collectivist		4	0.064	0.009	[−0.028, 0.155]	32.114^***^	90.658
**Grade level**	6.682						
Primary school		8	0.058	0.007	[−0.020, 0.135]	33.240^***^	78.941
Middle school		12	0.073	0.004	[0.022, 0.124]	45.821^***^	75.994
High school		1	0.150	0.000	[0.066, 0.232]	0.000	0.000
**Study attributes**							
**Publication type**	3.970^*^						
Journal		19	0.082	0.003	[0.043, 0.121]	89.562^***^	79.902
Doctoral dissertation		3	−0.129	0.033	[−0.322, 0.075]	1.092	0.000
**Intrusive parental homework involvement (IPI)**							
**Measuring tools**							
**Type of IPI**	0.004						
Controlling		10	−0.154	0.018	[−0.259, −0.045]	135.507^***^	93.358
Interference		9	−0.149	0.018	[−0.260, −0.034]	161.821^***^	95.056
**Questionnaire reporter**	0.327						
Students		12	−0.183	0.014	[−0.274, −0.089]	196.375^***^	94.398
Parents		7	−0.098	0.027	[−0.238, 0.046]	93.107^***^	93.556
**Math achievement indicators**	1.420						
Standardized measurement		11	−0.108	0.023	[−0.228, 0.014]	226.109^***^	95.577
Non-standardized measurement		8	−0.198	0.010	[−0.281, −0.112]	67.928^***^	89.695
**Demographics**							
**Culture**	70.039^***^						
Individualism		18	−0.193	0.009	[−0.257, −0.128]	222.015^***^	92.343
Collectivist		1	0.757	0.000	[0.617, 0.851]	0.000	0.000
**Grade level**	21.041^***^						
Primary school		10	−0.093	0.029	[−0.228, 0.046]	232.716^***^	96.133
Middle school		4	−0.228	0.005	[−0.300, −0.153]	15.680^**^	80.868
High school		1	−0.360	0.000	[−0.432, −0.284]	0.000	0.000
**Study attributes**							
**Publication type**	0.994						
Journal		18	−0.158	0.012	[−0.232, −0.081]	295.303^***^	94.243
Doctoral dissertation		1	−0.053	0.000	[−0.241, 0.139]	0.000	0.000

^*^p < 0.05; ^**^p < 0.01; ^***^p < 0.001.

**Table 4:** The correlation between parental homework involvement and students' mathematics achievement: Univariate regression analysis of continuous variables (random-effects model).

	**Variable**	**Parameter**	**Estimate**	**SE**	***Z-value***	**95% CI for *b***
	***Study attributes***					
SPI	*Publication year*	*β_0_*	0.0791	0.0203	3.89	[0.0393, 0.1190]
		*β_1_*	−0.0104	0.0061	−1.71	[−0.0224, 0.0015]
		*Q_*Model*_* (1, k = 22) = 2.94, *p* = 0.086				
IPI	*Publication year*	*β_0_*	−0.1480	0.0409	−3.62	[−0.2281, −0.0679]
		*β_1_*	0.0080	0.0087	0.92	[−0.0091, 0.0251]
		*Q_*Model*_* (1, k = 19) = 0.84, *p* = 0.358				

SPI, supportive parental homework involvement; IPI, intrusive parental homework involvement; *k* is the sample size of the independent study.

#### 3.4.1. Measuring tools

##### 3.4.1.1. Type of SPI/IPI

The homogeneity tests results showed that three different sub-types of supportive parental homework involvement can significantly moderate the relationship between SPI and students' mathematics achievement (*Q*__*BET*_SPI_ = 6.216, *df* = 2, *p* = 0.045), while two sub-types of intrusive parental homework involvement had no moderating effect on the relationship between IPI and students' mathematics achievement (*Q*__*BET*_IPI_ = 0.004, *df* = 1, *p* = 0.950). Specifically, when SPI was measured as autonomy support, content support, and provision of structure, respectively, the correlation between SPI and students' mathematics achievement decreased successively and even showed a weak negative correlation when measured as the provision of structure (*r*_SPI − AS_ = 0.133, 95% CI = [0.084, 0.181]; *r*_SPI − CS_ =0.049, 95% CI = [−0.002, 0.099]; *r*_SPI − PS_ = −0.009, 95% CI = [−0.243, 0.227]).

##### 3.4.1.2. Questionnaire reporter

The homogeneity test results showed that the questionnaire reporter has no moderating effect on the relationship between both SPI-students' math achievement link and IPI-students' math achievement (*Q*__*BETS*_PI_ = 2.293, *df* = 1, *p* = 0.084; *Q*__*BET*_IPI_ = 0.962, *df* = 1, *p* = 0.327).

##### 3.4.1.3. Mathematics achievement indicator

The homogeneity test results showed that it can significantly moderate the relationship between SPI and students' mathematics achievement (*Q*__*BET*_SPI_ = 14.423, *df* =1, *p* = 0.009), but has no effect on the relationship between IPI and students' mathematics achievement (*Q*__*BET*_IPI_ = 1.225, *df* = 1, *p* = 0.233). When students' mathematics achievement was indicated by non-standardized measurement, the correlation was stronger than indicated by standardized measurement (*r*_SPI-non-standardized_= 0.123, 95% CI = [0.087, 0.159], *r*_SPI-standardized_ = 0.036, 95% CI = [−0.019, 0.091]).

#### 3.4.2. Demographic variables

##### 3.4.2.1. Culture

Homogeneity test results showed that although cultural background could not moderate the relationship between SPI and students' mathematics achievement (*Q*__*BET*_SPI_= 0.088, *df* = 1, *p* = 0.767), it could significantly moderate the relationship between IPI and students' mathematics achievement (*Q*__*BET*_IPI_= 70.039, *df* = 1, *p* < 0.0001). However, given that the collectivist category included only one independent sample, we supposed that this moderating effect was not representative.

##### 3.4.2.2. Grade level

Homogeneity test results indicated that it could not significantly moderate the relationship between SPI and students' mathematics achievement (*Q*__*BET*_SPI_ = 6.682, *df* = 3, *p* = 0.083), but it could significantly moderate the relationship between IPI and students' mathematics achievement (*Q*__*BET*_IPI_ = 21.041, *df* = 3, *p* < 0.0001). To be more specific, with the increase in the grade level, the correlation between IPI and students' math achievement was gradually increasing (*r*_IPI-primary_ < *r*_IPI-middle_ < *r*_IPI-high_: −0.093 < −0.228 < −0.360).

#### 3.4.3. Study attributes

##### 3.4.3.1. Publication type

Homogeneity test results showed that it has a moderating effect on the relationship between SPI and students' math achievement (*Q*__*BET*_SPI_ = 3.970, *df* = 1, *p* = 0.046); but no moderating effect between IPI and students' math achievement (*Q*__*BET*_IPI_ = 0.994, *df* = 1, *p* = 0.319). However, considering that the source of 22 SPI studies only includes one doctoral dissertation (three independent samples from the dissertation were actually all from Nwokedi (2020) doctoral dissertation), we supposed that this moderation effect of publication type was not representative.

##### 3.4.3.2. Publication year

The results of the meta-regression analysis show that the publication year has no moderating effect on the relationship between SPI, IPI, and students' math achievement (*Q*_Model_ [1, k = 22] = 2.94, *p* = 0.086; *Q*_Model_ [1, k = 19] = 0.84, *p* = 0.358, respectively).

## 4. Discussion

This study analyzed the effects of 22 independent samples of SPI and 19 independent samples of IPI on students' mathematics achievement from 2005 to 2022. The results showed that SPI was significantly positively correlated with students' mathematics achievement, while IPI was significantly negatively correlated with students' mathematics achievement. Among them, the type of SPI, mathematics achievement indicators, and grade level moderated those effects.

### 4.1. Parental homework involvement and students' mathematics achievement

The results of meta-analysis support the hypotheses H1 and H2 that student's mathematics achievement was positively related to SPI and negatively related to IPI. These findings refute previous studies that reported non-significant or only negative correlations between parental homework involvement and math achievements (e.g., Karbach et al., 2013), demonstrating the value of supporting children's autonomy. As SDT states, autonomy, competence, and relatedness are three innate psychological needs of human beings, when they are satisfied, it yields enhanced self-motivation and mental health and when they are thwarted, it led to diminished motivation and wellbeing (Ryan and Deci, 2000). By enhancing students' feelings of autonomy, competence, and relatedness which contributes to their intrinsic motivation, SPI can improve students' mathematics achievement. In contrast, when parental homework involvement is intrusive, students' innate needs for competence, autonomy, and psychological relatedness were undermined (Moroni et al., 2015) and their persistence during homework tend to diminish, thus it may have a negative impact on their math achievement (Cooper et al., 2000; Grolnick and Pomerantz, 2009; Hill and Tyson, 2009; Dumont et al., 2012, 2014).

### 4.2. Moderation

The moderation tests showed that the link between SPI and students' mathematics achievement was moderated by three sub-types of SPI and mathematics achievement indicator, while the link between IPI and students' mathematics achievement was moderated by students' grade level; we will discuss these in the following subsections.

#### 4.2.1. Measuring tools

##### 4.2.1.1. Type of SPI

Among the three sub-types of SPI, the largest correlation was found between parental autonomy support and students' mathematics achievement. But a small positive correlation was found in content support-students' math achievement link, and even a negative correlation was found between the parental provision of structure-students' math achievement link, partially rejecting hypothesis H3-a. The largest correlation between parental autonomy support and students' math achievement is congruent with previous research (e.g., Viljaranta et al., 2018). Furthermore, it supports the SDT argument—autonomy support as a contextual factor plays a critical role in allowing individuals to actively satisfy all their needs. Satisfaction with each of the three psychological needs (autonomy, competence, and relatedness) is all facilitated by autonomy support (Ryan and Deci, 2017).

What needs to be carefully explained is the intriguing results that why parental content support showed a weak positive correlation with students' math achievement, and when measured as the provision of structure it even showed a weak negative correlation. One explanatory reason may be that parental content support, even when requested, may lead to a sense of incompetence for children (Xu et al., 2018; Xu and Corno, 2022). The sense of incompetence will lead to self-doubt, undermining children's self-efficacy and intrinsic motivation, and in turn reducing its positive impact on mathematical achievement. In addition, it is worth noting that although SDT indicated that parental provision of structure is critical in helping children develop a sense of control understanding and perceived competence, which become the basis for effective functioning (Grolnick and Ryan, 1989; Soenens et al., 2010), the premise is that students can internalize the values behind the activities supported by parents. However, students may display behavioral compliance by adapting their behavior to parental directives in the presence of the parental provision of structure but fail to internalize the values (Wang and Cai, 2017). For example, driven by Asian cultural values that emphasize interdependence and filial piety (Pomerantz et al., 2011; Cheung and Pomerantz, 2012), students are more inclined to display behavioral compliance to show their obedience, even though they do not agree with their parents' arrangement. Over time, they fail to internalize the values behind parental structural support or even have an aversion, but they never show it, which leads to their inability to develop control awareness, understanding, and perception, and ultimately has a negative impact on mathematics achievement. In addition, Ryan and Deci (2017) indicated that without autonomy support, the structure is not likely to be internalized to a degree that yields identified or integrated motivation. Furthermore, findings confirm that more beneficial outcomes occur under autonomy-supportive, high-structure circumstances (Grolnick et al., 2014). This provides inspiration for future parental homework involvement that a structuring parent is not one who just sets out rules and communicates consequences but who also facilitated the child in successfully enacting them and supports their autonomy as well.

##### 4.2.1.2. Mathematics achievement indicator

For students' mathematics achievement, non-standardized measurement showed a greater correlation in the SPI-mathematics achievement link, echoing Jeynes (2005) research, supporting hypothesis H5. When parents are supportively involved in students' homework and their support is perceived by teachers, it may affect the validity of teachers using non-standardized measurement to rate students' math achievement. As a result, students' mathematics achievement will become more positive, leading to a larger positive correlation between supportive parent homework involvement and students' mathematics achievement link.

#### 4.2.2. Grade level

In higher grade levels, IPI had stronger negative effects on students' mathematics achievement, supporting hypothesis H7-b. The moderating effect of grade level can be explained by the following aspects:

The first is the rising math anxiety of parents. This explanation was previously suggested by Maloney et al. (2015) that when higher-math-anxiety parents frequently help their children with math homework, their children learn less math over the course of the school year. Retanal et al. (2021) further proved that parents' math anxiety will have a negative impact on students' math achievement through parental intrusive homework involvement. On this basis, we can further deduce that the rising math anxiety of parents may be closely related to students' grade levels. As Hembree (1990) demonstrated that students' math anxiety varies in grade level: it is low or medium in primary school, and it then increases, peaks in the high school period, and slowly falls after graduation. For parents who involve in students' math homework, their anxiety may also differ across grade levels. To be more specific, the content of primary school mathematics homework is very basic, parent do not need to acquire expert knowledge and skills in mathematics to explain math problems in homework to their children (Szczygieł, 2020). However, with the increase in grade level, the math curriculum is more complex and abstract, and students start to have difficulties maintaining good performance in mathematics (Núñez et al., 2015). Correspondingly, parents may also feel more anxious when involved in advanced math homework, as they may lack sufficient knowledge and expertise (Jeynes, 2007; Patall et al., 2008; Wilder, 2014). In general, the increase in grade level drives the increase of parents' math anxiety, and parents' math anxiety will have an indirect negative impact on students' math achievement through IPI, which makes the negative correlation between IPI and students' math achievement show a trend of increasing with the grade level.

In addition, the mental characteristics of students in different grades can also explain the results. Compared to students in middle and high school, young children have less effective study habits and are less capable of avoiding distractions (Cooper and Valentine, 2001), thus parental control and interference are needed as an important way to help them focus and get rid of procrastination (Bronson, 2000). In contrast, middle- and high-school students have more developed self-regulation skills (Zimmerman and Pons, 1990), which supports them to become more autonomous, free, and independent, and conduct their learning in a more planned, conscious manner (Gorgoz and Tican, 2020). In this case, parents' control and interference will disrupt their rhythm by undermining their innate needs for competence, autonomy, and psychological relatedness. Thus, they had a stronger negative impact on middle and high school students' math achievement.

Culture and publication type show moderating effects on IPI-mathematics achievement and SPI-mathematics achievement link respectively. However, we believe that such moderating effects are caused by uneven sample size distribution and therefore are not representative. This inspires future meta-analyses to retest the moderating effect of these two variables on the basis of richer data. Meanwhile, the homogeneity test results showed that questionnaire reporters have no moderating effect. The result echoes Thomas et al. (2020), indicating a parallel between parent and student perception. Since many researchers believe that parents' and students' perceptions of what counts as parental involvement seem to vary (Barge and Loges, 2003; DePlanty et al., 2007), further studies are needed to shed light on the mixed results.

## 5. Implications

This meta-analysis has theoretical, practical, and methodological implications. The findings indicate that an ecological theoretical model is needed to understand the outcome of students' mathematics achievement (Bronfenbrenner, 1974). Whether students' autonomy is supported by parents' homework involvement, which is a type of interaction students experience in their immediate environment, plays an important role according to SDT theory (Ryan and Deci, 2000, 2017). The relationship between parental homework involvement and students' mathematics achievement is not an either-or issue. It is the type and quality of parental homework involvement that matters.

Practically, educators may utilize these findings to consider how to collaborate with parents in students' mathematics learning. First, schools can design and run family education workshops to increase parents' awareness of the value of autonomy support rather than just providing structural support, controlling, or interfering. Second, teachers may provide supportive counseling or direct strategies to help parents become more effectively involved in their children's homework, ensuring that instructional techniques parents use are in line with those being used by teachers. Third, teachers should use homework as a formative assessment tool to diagnose students' strengths and weaknesses in mathematics and improve instruction accordingly rather than just report summative scores to parents. It may reduce math anxiety of parents as grade level increases, and thus decrease instructive parental homework involvement and its negative impacts.

Methodologically, this meta-analysis showed the need to differentiate the type of parental homework involvement, mathematics achievement measurement, and grade level. Future studies should define different types of parental homework involvement more clearly and consider the impact of specific parental homework involvement types. Also, future studies should use standardized mathematics achievement tests to make the results more comparable. Furthermore, more longitudinal studies should be conducted to capture the differences across grade levels.

## 6. Limitations and prospects

Though this study followed meta-analysis methods and procedures, there are still some limitations in the classification of parental homework involvement, data collection, analysis of moderating variables, and selection of sample participants, which need to be improved in future research.

First, there is currently no comprehensive study on the classification of parental homework involvement, and questionnaires for each type of parental homework involvement are validated by the authors of included studies rather than standardized tests that have been widely used. Future studies should further classify parental homework involvement from a functional perspective and develop standardized scales to measure it. Second, in terms of data collection, this meta-analysis only included 41 independent samples. As more such studies accumulate, future meta-analysis might yield more profound results. In addition, we only examined the searchable literature published in English, thus future studies can expand the language range of literature search to Chinese, Japanese, Spanish, Korean, and so on. Third, regarding the analysis of moderating variables, there are significant differences in the sample size within some of the moderating variables examined in this study, which makes it difficult to ensure the robustness of the subgroup analysis results. Future research can further validate the analysis results of this study by enriching and balancing the number of studies within the moderating variable group. Finally, regarding the selection of sample groups, as the participants included were mainly focused on primary to high school students, future studies can include younger students (e.g., kindergarteners), school dropouts, or older adults.

## 7. Conclusion

This meta-analysis extends previous studies on the relationship between parental homework involvement and students' academic achievement with attention to types of parental involvement—supportive and intrusive, using mathematics as a specific subject. Through 41 effect sizes from 20 articles of 16,338 participants, we found a significant positive link between SPI and students' mathematics achievement and a negative link between IPI and students' mathematics achievement. The link between SPI and students' mathematics achievement differed across the three types of SPI (autonomy support, content support, and provision of structure) and mathematics achievement indicators. Specifically, autonomy support showed the strongest positive link, followed by content support and provision of structure. The link was stronger when measured by non-standardized measurements than standardized measurements. For the IPI-mathematics achievement link, it differed across students' grade levels, the negative link was strongest in high school, followed by middle school, and lowest in primary school.

## Data availability statement

The original contributions presented in the study are included in the article/Supplementary material, further inquiries can be directed to the corresponding author.

## Author contributions

QJ: writing-original draft preparation and methodology. LS: writing-reviewing, editing, and supervision. DZ: conceptualization, writing-reviewing, editing, and supervision. WM: methodology, supervision, and writing-reviewing and editing. All authors contributed to the article and approved the submitted version.

## Footnotes

^1^The codes of parents' homework involvement types for each independent sample were based on the questionnaire items used by the sample. For example, “My parent helped me find a quiet area for doing my 7th grade math homework (Nwokedi, 2020)” focuses on the structural support behavior of parents in the homework process, so it was coded as “provision of structure”; “My parents will not let me watch TV, or play with my friends…until I have finished my homework (Núñez et al., 2015)”. This item refers to pressure on students to complete homework, hence it was coded as “controlling”.

^2^Standardized measurement came exclusively from standardized math tests, while non-standard measurement involves some forms of teacher rating, school rating, and parents rating, such as math curriculum grades and school report card grades. This practice is common among existing meta-analyses on the topic (e.g., Jeynes, 2005; Ma et al., 2016).
